# Successful treatment of chronic alcohol-induced refractory hyponatremia with tolvaptan: a case report with in-depth analysis of traditional treatment failure mechanisms

**DOI:** 10.3389/fphar.2026.1794205

**Published:** 2026-05-11

**Authors:** Kui Yan, Jiang Zhou, Huanchao Zeng, Daiqiang Liu, Wentao Tao, Zhicheng Fang, Xianyi Yang

**Affiliations:** Department of Emergency Medicine, Taihe Hospital, Hubei University of Medicine, Shiyan, Hubei, China

**Keywords:** case report, chronic alcoholism, hyponatremia, syndrome of inappropriate antidiuretic hormone secretion, tolvaptan

## Abstract

**Background:**

Hyponatremia associated with the syndrome of inappropriate antidiuretic hormone secretion (SIADH) induced by chronic alcoholism exhibits high treatment resistance. The mechanisms underlying traditional treatment failure are often overlooked in clinical practice.

**Case Presentation:**

We report a case of a 59-year-old male patient with a 30-year history of heavy alcohol consumption, presenting with persistent serum sodium levels below 130 mmol/L for 3 months (nadir 116 mmol/L). Despite 3 months of aggressive sodium supplementation and fluid restriction therapy, serum sodium fluctuated between 116 and 125 mmol/L. Laboratory investigations revealed plasma ADH of 42 pg/mL (normal <5 pg/mL), plasma osmolality of 242 mOsm/kg, and urine osmolality of 486 mOsm/kg, consistent with SIADH diagnosis.

**Key Observations:**

Traditional treatment failure demonstrated a distinctive pattern: paradoxical increase in urine output during fluid restriction (intake 800 mL vs. urine output 1,200–1800 mL); paradoxical decrease in serum sodium following sodium supplementation (130→116 mmol/L) with compensatory increase in urinary sodium excretion (60→100 mmol/L); spironolactone increased urinary sodium excretion but serum sodium continued to decline.

**Intervention and Outcome:**

Treatment was switched to oral tolvaptan 15 mg once daily. Serum sodium increased to 128 mmol/L within 48 h and normalized to 135–138 mmol/L by day 7, with a correction rate of 8–9 mmol/L/24 h (within safe limits). No osmotic demyelination syndrome occurred. At 3-month follow-up, serum sodium remained stable within normal range.

**Novelty:**

This report provides the first systematic analysis of the specific resistance mechanisms of alcohol-induced SIADH to traditional treatment, revealing the molecular mechanisms underlying the “sodium paradox” (sodium supplementation paradoxically worsening hyponatremia), including V2 receptor upregulation leading to excessive water reabsorption, impaired hepatic ADH inactivation, and excessive compensatory renal sodium excretion.

**Conclusion:**

Tolvaptan demonstrates rapid, effective, and controllable therapeutic effects in alcohol-related refractory hyponatremia. This case emphasizes the importance of ADH measurement and pathophysiological mechanism analysis in such patients, providing a foundation for individualized treatment decisions.

## Introduction

Hyponatremia (serum sodium <135 mmol/L) is the most common electrolyte disturbance in clinical practice, occurring in 15%–30% of hospitalized patients (Lu et al., 2020). Patients with chronic alcoholism represent a high-risk population for electrolyte disorders, and the clinical manifestations of these disturbances are often atypical, leading to delayed diagnosis and treatment (Meenashi Sundaram et al., 2023). Unlike SIADH caused by small cell lung cancer or traumatic brain injury, alcohol-induced hyponatremia exhibits distinctive characteristics: (1) prolonged disease course typically spanning months to years; (2) limited benefit from traditional treatments (fluid restriction and sodium supplementation) (Martin-Grace et al., 2022); (3) frequently overlooked mechanisms of treatment failure, leaving clinicians without theoretical guidance; and (4) poor patient compliance with high recurrence rates.

Although vasopressin receptor antagonists (vaptans) provide a novel therapeutic option for refractory hyponatremia, their application strategies in complex alcohol-related SIADH and the understanding of traditional treatment failure require further exploration. Through systematic analysis of a typical case of alcohol-induced SIADH with complete traditional treatment failure, this report elucidates the specific pathophysiological barriers and demonstrates the rapid efficacy of tolvaptan.

## Case presentation

### Patient information

A 59-year-old Han Chinese male, retired worker, weighing 62 kg, was admitted in August 2025 with chief complaints of “fatigue, anorexia, and altered mental status for 1 week”

### Medical history

#### Alcohol use history

The patient had a 30-year history of heavy alcohol consumption, drinking approximately 500 mL of distilled spirits daily (equivalent to 50 g of pure ethanol). This chronic consumption pattern remained relatively stable over the 30-year period until his admission in August 2025, when he presented with acute clinical decompensation. He was diagnosed with “alcoholic liver disease” 3 years prior due to abnormal liver function but had not achieved strict abstinence.

#### Timeline of hyponatremia

**FIGURE 1:**
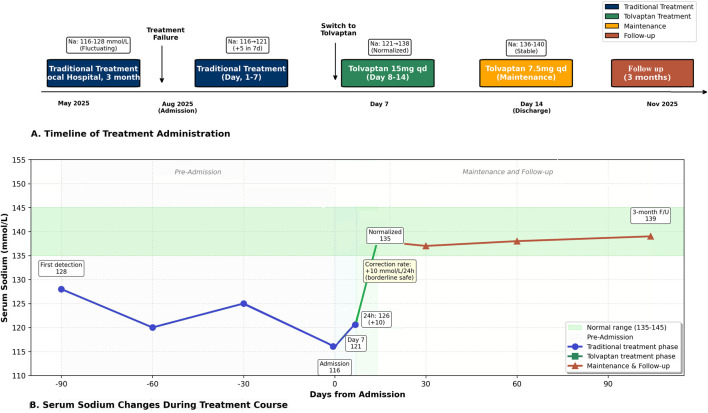
Treatment timeline and serum sodium response in alcohol-induced refractory SIADH. **(A)** Timeline of treatment administration from the episode of care. The patient received traditional treatment (fluid restriction, sodium supplementation, and spironolactone) for 3 months at a local hospital and 7 days after admission, with persistent hyponatremia. Treatment was switched to tolvaptan on Day 8, resulting in normalization of serum sodium by Day 14. **(B)** Serum sodium changes during the treatment course. Note the fluctuating sodium levels during traditional treatment (116–128 mmol/L) and the rapid, sustained correction following tolvaptan initiation. Green shaded area indicates normal range (135–145 mmol/L).

Serum sodium abnormality (128 mmol/L) was first detected in mid-May. Subsequently, the patient received intermittent aggressive treatment at a local hospital for 3 months, including oral sodium supplementation 3 g/day, intravenous 3% NaCl 250 mL once or twice daily, fluid restriction to 600–800 mL/day, and spironolactone 25–50 mg/day. However, serum sodium continued to fluctuate between 116 and 125 mmol/L with recurrent symptom deterioration (Figure 1).

#### Past medical history

Ten months prior, the patient sustained a left epidural hematoma from syncope-related fall, managed conservatively with complete resolution. He had a 20-year smoking history of one pack daily. He denied hypertension, diabetes mellitus, heart disease, or pulmonary conditions.

### Physical examination on admission

#### Vital signs

Temperature 36.8 °C, pulse 65 beats/min, respiratory rate 18 breaths/min, blood pressure 112/77 mmHg (normotensive).

#### Neurological examination

Alert but lethargic, delayed responses, disorientation to time and place. Pupils equal, round, and reactive. Meningeal signs negative.

#### General examination

No jaundice or significant edema. Cardiopulmonary auscultation unremarkable. Abdomen soft and non-tender.

### Laboratory investigations

#### Liver function

AST 92 U/L (↑), ALT 68 U/L (↑), AST/ALT ratio 1.35 > 1 (typical of alcoholic liver disease), albumin 32 g/L (↓, indicating impaired hepatic synthetic function), γ-GT 156 U/L (↑). Calculated APRI score = [(92/40)/(145)] × 100 ≈ 1.6, suggesting moderate hepatic fibrosis.

#### Other parameters

Vitamin B1 48 nmol/L (↓), Vitamin B12 178 pg/mL (↓). Thyroid function, cortisol levels, and immune markers were all within normal limits. No autoimmune markers detected (Table 1).

**TABLE 1:** Key laboratory findings on admission.

Parameter	Value	Reference range	Clinical significance
Serum sodium	116 mmol/L	135–145	Severe hyponatremia
Plasma osmolality	242 mOsm/kg	280–300	Hypotonic state
Urine osmolality	486 mOsm/kg	200–800	Inappropriately elevated
Urine sodium	68 mmol/L	20–250	Elevated despite hyponatremia
Serum creatinine	76 μmol/L	60–110	Normal renal function
Blood urea nitrogen	4.8 mmol/L	2.5–7.1	No dehydration
Plasma ADH	42 pg/mL	<5	Markedly elevated (>8× normal)

#### Diagnostic criteria compliance

The patient fully met the diagnostic criteria for SIADH: (1) hypotonic hyponatremia (242 mOsm/kg); (2) evidence of inappropriate vasopressin activity relative to plasma osmolality with inappropriately elevated urine osmolality (486 mOsm/kg); (3) clinical euvolemia; (4) urine sodium >30 mmol/L; (5) no recent diuretic use; (6) normal renal function; (7) exclusion of glucocorticoid deficiency and hypothyroidism (Verbalis et al., 2013; Bartter and Schwartz, 1967).

Imaging Studies: Head CT: Lacunar infarcts. Chest CT and abdominal CT: No significant abnormalities. Abdominal ultrasound: Coarsened liver echo pattern, consistent with alcoholic liver disease.

### Traditional treatment phase (Days 1–7)

#### Treatment regimen

Alcohol abstinence; fluid restriction to 800 mL/day; oral sodium chloride tablets 3 g three times daily (total 9 g/day); intravenous 3% NaCl 250 mL once daily (sodium supplementation 11 mmol/day); spironolactone 25–50 mg once daily; vitamin B1 100 mg and B12 500 μg once daily; hepatoprotective therapy with compound glycyrrhizin injection.

#### Treatment response

After 7 days, serum sodium increased to only 121 mmol/L (improvement of only 5 mmol/L). The patient’s symptoms showed no significant improvement, with persistent fatigue and lethargy.

### Tolvaptan treatment phase (Days 8–14)

#### Rationale for treatment switch

Following thorough discussion with the patient and family regarding informed consent, given the complete failure of 3 months of traditional treatment, the decision was made to switch to tolvaptan.

#### Treatment protocol

Tolvaptan 15 mg orally once daily in the morning; fluid restriction continued at 1,000 mL/day; B-vitamin supplementation and hepatoprotective therapy continued.

#### Monitoring

Serum sodium measured every 6–8 h during initial treatment to prevent overcorrection (Table 2).

**TABLE 2:** Dynamic changes during tolvaptan treatment.

Time point	Serum Na (mmol/L)	Daily Δ (mmol/L/24 h)	Urine output (mL/24 h)	Urine Osm (mOsm/kg)	Clinical status
Baseline	116	-	1,200	486	Lethargic, disoriented
12 h	122	-	1800	350	Initial improvement
24 h	126	+10	2,400	250	Symptoms improving
48 h	128	+2	2,200	240	Mental status improved
72 h	132	+4	1800	230	Markedly improved
Day 7	135	+3.4*	1,600	200	Asymptomatic
Day 14	138	+0.4*	1,400	185	Ready for discharge

* Average daily change from Day 4 onward.

#### Adverse event monitoring

During tolvaptan treatment, the patient experienced no significant adverse effects such as thirst, dry mouth, or urinary frequency. Liver enzymes showed no significant elevation compared to baseline (ALT 71 U/L, AST 95 U/L), and renal function remained stable (creatinine 74 μmol/L, urea 4.2 mmol/L). No clinical manifestations or neurological deterioration suggestive of osmotic demyelination syndrome were observed.

### Discharge and follow-up

#### Discharge status (Day 14)

Serum sodium 135–138 mmol/L, general condition good, neurological symptoms resolved, mental status clear, appetite recovered, able to perform activities of daily living independently.

#### Discharge instructions

(1) Strict alcohol abstinence (most critical); (2) Tolvaptan 7.5 mg orally once daily (maintenance dose reduction); (3) B-complex vitamin supplementation; (4) Regular electrolyte monitoring (at 1, 2, 4, and 8 weeks post-discharge).

#### Follow-up results (3 months post-discharge)

Patient maintained alcohol abstinence. Serum sodium remained at 136–140 mmol/L (ideal range). Liver enzymes showed mild improvement. No symptom recurrence. Patient expressed high satisfaction with treatment.

## Discussion

### Diagnostic precision of SIADH

This patient presented with both direct plasma ADH measurement (42 pg/mL, markedly elevated) and complete diagnostic criteria compliance, providing high diagnostic certainty. Key diagnostic evidence included: (1) hypotonic plasma (242 vs. 280–300 mOsm/kg); (2) inappropriately elevated urine osmolality (486 vs. expected <100 mOsm/kg in hypotonic states); (3) clinical euvolemia without hypoperfusion; (4) urine sodium concentration >30 mmol/L; (5) no recent diuretic use; (6) normal renal function; (7) normal thyroid and adrenal function; (8) ADH level remaining >5 pg/mL despite hypotonic state.

SIADH was first described in 1950 in two patients with lung cancer (Schwartz et al., 1957). Currently, SIADH has been associated with numerous conditions (Martin-Grace et al., 2022; Adrog et al., 2000; Melmed et al., 2020) (Table 3). This patient’s history, clinical presentation, laboratory findings, and imaging studies excluded common causes of SIADH. Therefore, inappropriate ADH secretion in this patient was attributed to chronic alcohol toxicity from prolonged heavy drinking. However, due to financial constraints, genetic testing for V2 receptor activating mutations was not performed, representing a relative limitation.

**TABLE 3:** Causes of syndrome of inappropriate antidiuresis.

Causes of SIAD
Malignancy
Lung cancer
CNS tumors
Head and neck tumors
Hematological
Urological
Gastrointestinal
Central nervous system
Trauma
Vascular insult (SAH, SDH)
Infection (meningitis, encephalitis)
Inflammation
Medications
Chemotherapy
Psychiatric
Other: Omeprazole, nicotine, oxytocin, clofibrate, carbamazepine
Respiratory
Infectious: Pneumonia, TB, empyema
Mechanical: COPD, acute respiratory failure, positive pressure ventilation
Other
Nausea
Pain
Prolonged exercise
Surgery
Activating mutations of V2 receptor
Idiopathic

Abbreviations: CNS, central nervous system; COPD, chronic obstructive pulmonary disease; SAH, subarachnoid hemorrhage; SDH, subdural hematoma; SIAD, syndrome of inappropriate antidiuresis; TB, tuberculosis; V2 receptor,vasopressin-2, receptor.

### Multi-level mechanisms of traditional treatment failure

The treatment failure pattern in this patient revealed specific pathophysiological barriers in alcohol-induced SIADH.

#### Failure of fluid restriction (“urine volume paradox”)

Clinical observation: With fluid restriction to 800 mL/day, urine output paradoxically increased to 1,200–1800 mL/day (expected negative fluid balance), while serum sodium declined rather than increased.

Pathophysiological mechanisms: (1) V2 receptor saturation activation: Under extremely elevated ADH (42 pg/mL) and sustained high ADH state for 3 months, renal collecting duct V2 receptors are 100% saturated. Even with ADH reduction, at the plateau of the saturation curve, renal response to ADH signaling remains unchanged (Kim et al., 2021); (2) Osmoreceptor dysfunction: Normally, plasma osmolality of 242 mOsm/kg should completely suppress ADH secretion, but this patient maintained ADH at 42 pg/mL, indicating destruction of hypothalamic osmoreceptor sensitivity and feedback mechanisms to hypotonic stimuli (Liamis et al., 2000); (3) Adaptive cerebrospinal fluid regulation: Under chronic hypotonic conditions, cerebrospinal fluid solute concentration has adapted accordingly, and the plasma osmolality increase induced by fluid restriction is insufficient to trigger effective ADH response (Liamis et al., 2000).

#### Failure of sodium supplementation (“sodium paradox”)

Clinical observation: After 3 months of sodium supplementation (combined oral and intravenous), serum sodium paradoxically decreased from 130 to a nadir of 116 mmol/L; simultaneously, urinary sodium increased (60→100 mmol/L).

Pathophysiological mechanisms (triple barrier): (1) Excessive compensatory urinary sodium excretion: Supplemented sodium → transient plasma osmolality increase → renal detection → compensatory excretion increase (>supplementation amount) → net result: supplemented sodium completely or partially excreted. Data from this case support this: Sodium supplementation approximately 150 mmol/day, but urinary sodium increased from 60 to 100 mmol/L, reaching 67%–100% of supplementation. This degree of compensation indicates that renal sodium handling is subject to abnormal “dilution drive” (Winzeler et al., 2016); (2) High ADH-driven water reabsorption dominance: Even when compensatory urinary sodium excretion cannot completely consume supplemented sodium, high ADH continues to drive water reabsorption. The interaction between supplemented sodium and continuously reabsorbed water ultimately results in relative dilution of serum sodium. This reflects that in SIADH, the water problem (ADH-driven excessive reabsorption) masks sodium increases (Warren et al., 2023); (3) Impaired hepatic ADH inactivation: The patient’s liver score (APRI 1.6) suggests moderate hepatic fibrosis. Literature supports that the liver should inactivate 80%–90% of circulating ADH, but in patients with moderate liver damage, this capacity can decrease by 40%–50%. Even if sodium supplementation induces some compensatory ADH reduction, insufficient hepatic inactivation maintains ADH at the elevated level of 42 pg/mL (Praharaj and Anand, 2022).

#### Limitations of spironolactone therapy

Spironolactone, as an aldosterone antagonist, acts on the distal tubule and early collecting duct. Although it increased urinary sodium excretion (60→100 mmol/L), serum sodium paradoxically decreased (120→118 mmol/L), indicating that excreted sodium failed to effectively elevate serum sodium concentration. The reason is that the fundamental problem of SIADH (excessive water reabsorption) is not at spironolactone’s site of action; high ADH continues to act in the late collecting duct, resulting in sodium excretion but water retention (Liamis et al., 2000).

### Mechanism and advantages of tolvaptan

Tolvaptan is a selective vasopressin V2 receptor antagonist with a mechanism of action fundamentally different from traditional treatments (Lewellyn et al., 2025). Site of action: Direct blockade of V2 receptors on renal collecting duct epithelial cells, interrupting ADH signaling pathway and preventing aquaporin-2 (AQP2) membrane expression. Expected effects: Promotion of “free water” excretion while maintaining normal serum sodium and sodium metabolism, without increasing urinary sodium loss.

Actual observations in this case: (1) Increased urine output: From 1,200 to 1,600 mL/24 h (+33%) within 6 h of administration, peak of 2,400 mL (+100%) at 24 h, confirming reduced water permeability due to decreased AQP2 expression; (2) Rapid decline in urine osmolality: From 486 mOsm/kg to 250 mOsm/kg at 24 h, further confirming excretion of dilute rather than concentrated urine; (3) Steady serum sodium increase: +10 mmol/L in the first 24 h (borderline but safe), subsequently +2–4 mmol/L daily (actively controlled gradual curve), completely different from the “stagnation or decline” seen with traditional treatment (Table 4).

**TABLE 4:** Fundamental differences between tolvaptan and traditional treatment.

Aspect	Traditional treatment	Tolvaptan
Target	Fluid intake, sodium supplementation, distal tubule	V2 receptor, AQP2
Renal response	Continued water retention (maintained high urine output)	Water excretion (increased urine output)
Urine osmolality	Elevated or unchanged (concentrated urine)	Decreased (dilute urine)
Serum sodium change	Stagnation or decline	Steady increase
Pathology addressed	Attempts to overcome ADH (fails)	Bypasses ADH signaling (succeeds)

This mechanistic difference explains why “intensifying traditional treatment” cannot succeed, while changing the target (tolvaptan) produces immediate effects.

### Strict management of ODS risk

Although this patient had severe and prolonged hyponatremia, osmotic demyelination syndrome (ODS) did not occur, attributable to three factors (Warren et al., 2023): (1) Strict control of correction rate: +10 mmol/L in the first 24 h (at threshold but not exceeded), followed by immediate deceleration to +2–4 mmol/L/day (well below the safety limit of 6 mmol/L/day for chronic hyponatremia). Cumulative average of 3.4 mmol/L/day, compliant with guidelines; (2) Continuous neurological monitoring: Daily examination revealed no new pyramidal tract signs, oculomotor abnormalities, or altered consciousness suggestive of ODS. Pre-existing disorientation improved significantly after day 3 of sodium correction, correlating with serum sodium improvement; (3) Case-specific low ODS risk features: In pure SIADH (non-hypovolemic type) patients, cerebrospinal fluid has already adapted to hypotonic conditions, and the rebound mechanism during correction is less severe than in hypovolemic patients.

### Uniqueness of alcohol-induced SIADH

Compared with other SIADH types, this case demonstrates the specific characteristics of alcohol-related disease. Triple barrier theory: (1) Central nervous system barrier: Toxic damage to hypothalamic osmoreceptors prevents ADH suppression by hypotonic stimuli; (2) Hepatic barrier: Hepatic fibrosis leads to decreased ADH inactivation; (3) Renal barrier: Chronic high ADH causes V2 receptor upregulation, making kidneys hypersensitive to ADH. Unlike lung cancer-type SIADH (pure ADH hypersecretion) (Martin-Grace et al., 2022) or traumatic brain injury-type SIADH (may be self-limiting) (Lee et al., 2023), alcohol-induced SIADH will persist without fundamental intervention (abstinence) due to continuation of these triple barriers.

### Clinical implications

#### Diagnostic precision

Direct ADH measurement should be performed in patients with refractory hyponatremia, rather than relying solely on indirect diagnosis based on “low plasma osmolality + high urine osmolality.” This patient’s ADH of 42 pg/mL directly confirmed the diagnosis.

#### Individualized treatment decisions

If ADH >30 pg/mL and cannot be suppressed by hypotonic stimuli → tolvaptan preferred; if sodium supplementation ineffective for 3 months → target change needed; if liver disease present → tolvaptan’s high V2 selectivity advantageous (avoiding V1-mediated vascular/platelet effects).

#### Fundamental treatment

Abstinence is not “adjunctive” but essential. Even with serum sodium correction to normal, continued alcohol consumption will cause SIADH recurrence, with recurrence rates >80%. As emphasized in recent comprehensive analyses of alcohol abuse disorder, the systemic consequences extend beyond electrolyte abnormalities to include progressive hepatic fibrosis, cardiomyopathy, and neurological deterioration (Argo et al., 2024). Therefore, pharmacological management of hyponatremia must be coupled with robust behavioral intervention and addiction medicine support to address the underlying etiology and prevent recurrence. This patient’s sustained abstinence at 3-month follow-up, resulting in stable serum sodium levels without recurrence, underscores the critical importance of this fundamental treatment modality.

## Conclusion

This report emphasizes the importance of precise diagnosis, mechanism analysis, and individualized treatment in patients with alcohol-related refractory hyponatremia. Tolvaptan represents a critical alternative when traditional treatment fails. Importantly, we underscore that abstinence and behavioral intervention constitute fundamental treatment—not merely adjunctive measures—essential for preventing SIADH recurrence and addressing the broader pathophysiological consequences of chronic alcohol toxicity. The sustained clinical success achieved in this case is attributable to both the efficacy of tolvaptan in correcting the acute electrolyte abnormality and the patient’s commitment to alcohol abstinence, which eliminates the underlying pathogenic stimulus.

## Data Availability

The original contributions presented in the study are included in the article/supplementary material, further inquiries can be directed to the corresponding authors.
